# Double dose alglucosidase-alpha doubles benefit?

**DOI:** 10.1016/j.ymgmr.2018.07.004

**Published:** 2018-07-20

**Authors:** Josef Finsterer, Fulvio A. Scorza, Ana C. Fiorini, Carla A. Scorza, Antonio Carlos Almeida

**Affiliations:** a Krankenanstalt Rudolfstiftung, Vienna, Austria; b Disciplina de Neurociência, Escola Paulista de Medicina/Universidade Federal de São Paulo/. (EPM/UNIFESP), São Paulo, Brazil; c Programa de Estudos Pós-Graduado em Fonoaudiologia, Pontifícia Universidade Católica de São Paulo (PUC-SP), Departamento de Fonoaudiologia, Escola Paulista de Medicina/Universidade Federal de São Paulo (EPM/UNIFESP), São Paulo, Brazil; d Laboratory of Experimental and Computational Neuroscience, Department of Biosystems Engineering, Brazil

We read with interst the article by Landis et al. about a 3 monthsold male with dilated cardiomyopathy due to infantile onset Pompe disease (IOPD) who profited from increased dosage of algucosidase-alpha (40 mg/kg/d twice a week) [1]. We have the following comments and concerns.

Echocardiography in fig. 3 (axial view), particularly panel d and e, suggests that the patient had left ventricular hypertrabeculation / noncompaction (LVHT) [2]. Was he ever been sepcifically investigated for this abnormality by transesophageal echocardiogrpahy (TEE), ventriculography, or cardiac MRI? LVHT has not been reported in IOPD so far.

Most of the clinical manifestations in the index patient improved upon enzyme replacement therapy (ERT) [1]. Did also dysphagia improve upon ERT? Was the patient able to eat by himself at the last follow-up or did he still require the gastrostomy tube? Did he also present with dysarthria at onset?

Patients wirh Pompe disease may also develop dilative arteriopathy, particularly of the cerebral arteries [3]. Did the index case undergo computed tomography angiography (CTA) or magnetic resonance angiography (MRA) to exclude or confirm dilated arteriopathy? Did dilated artiopathy improve upon the higher ERT dosage?

Was muscle hypotonia attributable to involvement of the brainstem, the peripheral nerves, or the muscles? Did he ever undergo nerve conduction studies (NCSs) or needle electromyography (EMG)? Was respiratory function ever tested?

Was the diagnosis IOPD genetically confirmed in the index case? Was the mother tested for the mutation? Did she also manifest with clinical features of Pompe disease? Though autosomal recessive, it is conceivable that heterozygotes manifest clinically as well.

Overall, the presented case could be more meaningful if supplementary data about the phenotype and genotype of the mother would have been provided, if cardiac imaging would heve been revised for LVHT, and if the index case would have been screened for dilative arteriopathy.

## Conflicts of interest

There are no conflicts of interest.

## Funding

No funding was received.

## Author contribution

JF: design, literature search, discussion, first draft, FS, AF, CS, ACA: literature search, discussion, critical comments.
